# Diet, life-style and cardiovascular morbidity in the rural, free living population of Elafonisos island

**DOI:** 10.1186/s12889-017-4053-x

**Published:** 2017-02-01

**Authors:** Chris J. Kapelios, Ioannis Kyriazis, Ioannis Ioannidis, Charilaos Dimosthenopoulos, Erifili Hatziagelaki, Stavros Liatis, The PERSEAS Study Group, Emmanouil Mpelliotis, Ioannis Papadopoulos, Filitsa Spanoudi, Konstantinos Sgouros, Dimitris Logothetis, Eirini Papaneofytou, Gerasimos Markatos, Stamatia Moschakou, Dimitrios Mpramos, Ekaterini Konstantatou, Georgios Antonakoudis, Matina Nezi, Matina Kleftaki, George Markozannes

**Affiliations:** Hellenic Medical Society for the study of Risk Factors in Vascular Diseases, 8 Iak. Dragatsi Street, 18535 Peiraias, Greece

**Keywords:** Cardiovascular disease, Mediterranean diet, Obesity, Cardiovascular risk, Diabetes mellitus, Hypertension

## Abstract

**Background:**

There are about 70 small islands in the Aegean and Ionian Sea, of less than 300 Km^2^ and 5000 inhabitants each, comprising a total population of more than 75,000 individuals with geographical and socioeconomic characteristics of special interest. The objective of the present study was to assess lifestyle characteristics and the state of cardiovascular risk of the population of a small Eastern Mediterranean island, Elafonisos.

**Methods:**

PERSEAS (Prospective Evaluation of cardiovascular Risk Surrogates in Elafonisos Area Study) is an ongoing, population-based, longitudinal survey of cardiovascular risk factors, life-style characteristics and related morbidity/mortality performed in a small and relatively isolated island of the Aegean Sea, named Elafonisos. Validated, closed-ended questionnaires for demographic, socio-economic, clinical and lifestyle characteristics were distributed and analyzed. The MedDietScore, a validated Mediterranean diet score was also calculated. In addition, all participants underwent measurement of anthropometric parameters, blood pressure and a full blood panel for glucose and lipids.

**Results:**

The analysis included 596 individuals who represented 74.5% of the target population. The mean age of the population was 49.5 ± 19.6 years and 48.2% were males. Fifty participants (8.4%) had a history of cardiovascular disease (CVD). The rates of reported diabetes, hypertension, and hypercholesterolemia were 7.7%, 30.9% and 30.9% respectively, with screen-detection of each condition accounting for an additional 4.0%, 12.9%, and 23.3% of cases, respectively. Four hundred and seven individuals (68.3%) were overweight or obese, 25% reported being physically inactive and 36.6% were active smokers. The median MedDietScore was 25 [interquartile range: 6, range 12–47] with higher values significantly associated with older age, better education, increased physical activity, absence of history of diabetes and known history of hypercholesterolemia.

**Conclusions:**

Obesity and traditional risk factors for CVD are highly prevalent among the inhabitants of a small Mediterranean island. Adherence to the traditional Mediterranean diet in this population is moderate, while physical activity is low. There seems to be a need for lifestyle modification programs in order to reverse the increasing cardiovascular risk trends in rural isolated areas of the Mediterranean basin.

**Electronic supplementary material:**

The online version of this article (doi:10.1186/s12889-017-4053-x) contains supplementary material, which is available to authorized users.

## Background

Over the past half century, large, population-based, epidemiologic studies, among which the Framingham Heart Study represents a landmark [1], have established the prognostic significance of several factors such as hypercholesterolemia, arterial hypertension (AH) and diabetes mellitus (DM) in the appearance of cardiovascular morbidity and mortality [2, 3]. Further investigations demonstrated the etiologic association between the aforementioned morbid conditions and the incidence of cardiovascular disease (CVD), in this way recognizing the former as potential therapeutic targets [4, 5]. Despite the undisputed efficacy and safety of several medications aiming to decrease cardiovascular risk, it has been reported that CVD: a) remains the leading cause of mortality, accounting for approximately one third of all deaths worldwide, b) claims more lives than all cancers combined and c) accounts for annual direct and indirect costs in excess of $ 316.6 billion in the U.S.A. alone [6].

Despite the lack of relevant collective consciousness and education, the importance of behavior-modifying measures in improving health and prolonging survival is paramount [7]. The Mediterranean diet represents a typification of a balanced intake of nutrients, vitamins and traces, while its association with improvement of several health indices has been stressed by various studies [8–10]. The qualitative composition of the diet, however, varies greatly among Mediterranean countries and within regions of the same country [11].

There are in Greece about 70 small islands spread in the Aegean and Ionian seas, of less than 300 km^2^ and 5000 inhabitants each, representing a total population of about 75,000 individuals, with geographical and socioeconomic characteristics of special interest. Islands may serve as “epidemiologic laboratories” in which different hypotheses can be tested that could otherwise not be readily assessed elsewhere [12]. Greek island inhabitants have been traditionally occupied with agricultural, farming and piscatorial activities and are considered to be populations highly adherent to the Mediterranean diet. The classic Seven Country Study (SCS), which demonstrated the significance of traditional Mediterranean diet in limiting the incidence of coronary heart disease during the 1950’s, included individuals from two big Greek islands (Crete and Corfu) [13]. Nonetheless, no systematic investigation of dietary and life-style habits in the very small islands and their association with the presence of CVD has ever been performed.

**Fig. 1:**
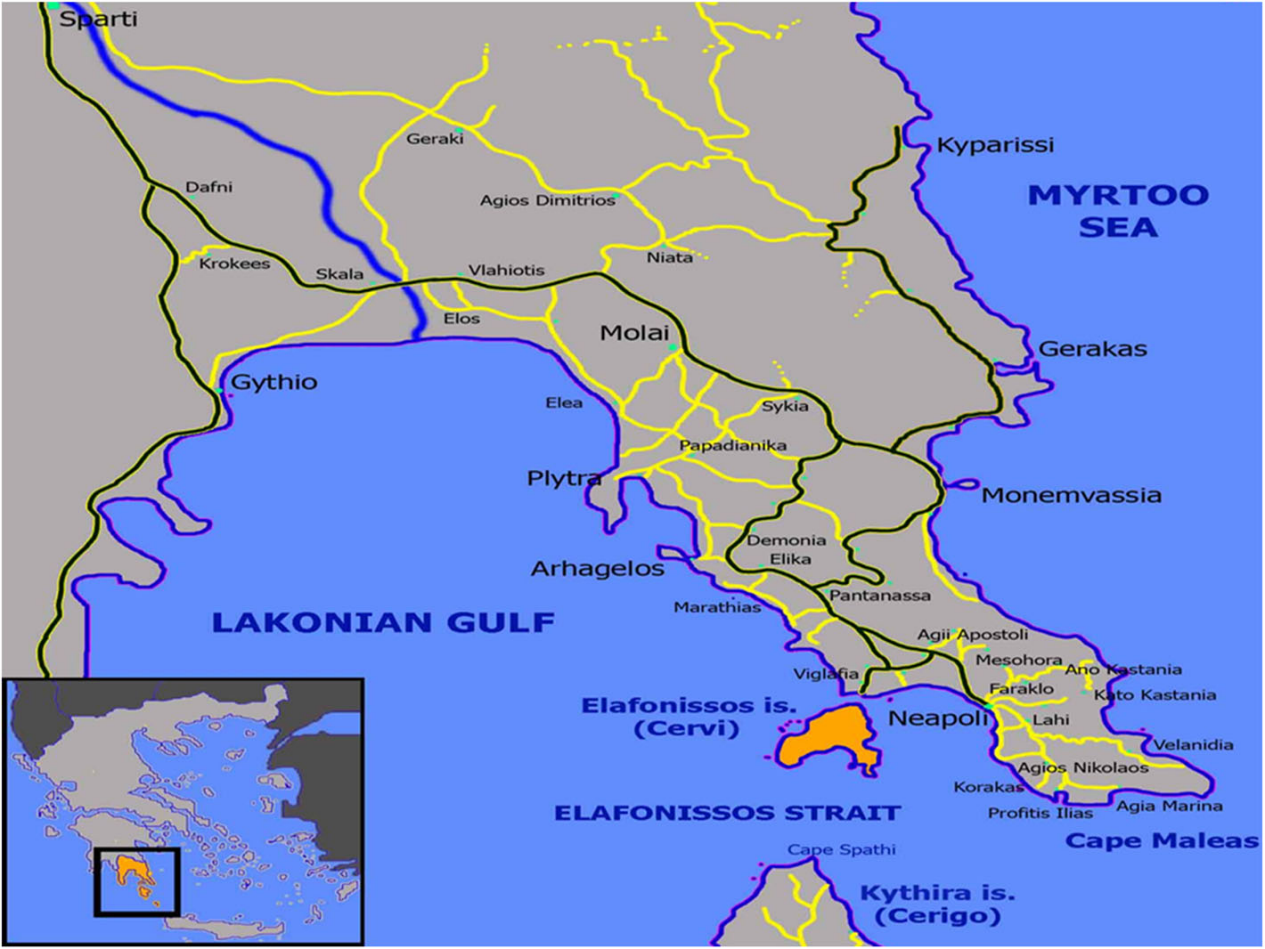
Map of Elafonisos. The island is highlighted orange

PERSEAS (Prospective Evaluation of cardiovascular Risk Surrogates in Elafonisos Area Study) is an ongoing, population-based, longitudinal survey of cardiovascular risk factors, life-style characteristics and related morbidity/mortality in Elafonisos, a small, relatively isolated island of about 20 km^2^ and about 1000 inhabitants (Fig. 1). In the present analysis we report the main results of the baseline assessment of PERSEAS, performed during 2012 and 2013. More specifically, we aimed to assess a) the prevalence of established CVD; b) the prevalence of traditional risk factors for CVD (diabetes mellitus, hypertension, hypercholesterolemia); c) dietary and lifestyle habits, and d) factors associated with adherence to the traditional Mediterranean diet.

## Methods

### Study population

The target population consisted of all permanent, inhabitants of Elafonisos island > 15 years of age during the study period. The analysis included participants who were interviewed and underwent full clinical evaluation. The reference population (total number of the island adult inhabitants) was defined according to the 2011 census of the Greek population by the National Statistics Agency [http://www.statistics.gr/en/2011-census-pop-hous, last accessed on 09/01/2016]. The interview and clinical examinations were performed by trained personnel during two pre-specified weeks in September 2012 and September 2013 respectively.

### Study questionnaires

The basic questionnaire included information on a wide variety of features including demographics, educational level, smoking habits, sleep habits and a detailed medical history.

Dietary habits were assessed using a validated food-frequency questionnaire. The questionnaire was validated regarding its test-retest reliability in our population with a random sub-group of 50 individuals who completed the questionnaire twice with a two week interval. Pearson’s correlation coefficient was 0.88 (*P* < 0.001). The individuals answered questions about their typical weekly consumption of different food categories (whole grains, fruits, vegetables, potatoes, legumes, fish, olive oil, red meat, poultry and full fat dairy products). Weekly alcohol consumption was estimated on the basis of 100 ml-wine glass equivalents [14]. A validated dietary score assessing adherence to the traditional Mediterranean diet, the MedDietScore [14], which has been previously described in detail [15, 16], was calculated. In brief, the score takes values between 0 and 55 with greater values indicating higher adherence. The exact scoring according to the frequency of consumption of every food category is depicted in in Table 1 of the Additional file 1.

The level of physical activity was assessed by answering to closed-ended questions regarding the usual type of activity (walking, jogging, sport), the frequency of activity (none, daily, less or more often than three times per week) and its mean duration.

### Anthropometric measurements and blood pressure

All participants underwent height and weight measurements on site. An average of two measurements of height was obtained with a calibrated anthropometer, used by trained personnel. The participants were weighed without shoes, wearing light clothing and, subsequently, the two values were averaged. Body weight was determined to the nearest 100 g using a digital scale. Body mass index (BMI) was calculated by bodyweight in kilograms divided by height in meters squared. The patients were divided into BMI categories (underweight, normal weighted, overweight and obese according to the World Health Organization (WHO) classification [17]. Waist circumference, measured at the midpoint between the costal margin and iliac crest in the mid-axillary line, hip circumference, measured at the tip of the hip bone in men, and at the widest point between the hips and the buttocks in women and neck circumference, measured in the midway of the neck, between mid-cervical spine and mid anterior neck were also assessed using a non-stretchable, plastic, measuring tape and rounded to the nearest 0.1 cm. Body fat was measured using electrical bio-impedance (Body Composition Analyzer SC-330, Tanita). Lean body mass was calculated by subtracting body fat from total body weight.

Blood pressure was measured according to the standards outlined in the relevant 2013 European Society of Hypertension/European Society of Cardiology guidelines on AH [18]. Specifically, blood pressure was measured by use of a validated electronic device (M2 Basic Upper Arm Blood Pressure Monitor, Omron). Hypertension was defined as systolic blood pressure ≥140 mmHg and/or diastolic blood pressure ≥90 mmHg. The diagnosis of hypertension was based on two blood pressure measurements in the sitting position. Patients with elevated values of BP were re-evaluated on a second visit.

### Blood measurements

All blood measurements were performed in the morning, after an eight-hour, overnight fast. A validated point-of-care portable analyzer was used to measure plasma lipids and glucose levels through a combination of enzymatic methodology and solid-phase technology (CholestechLDX®Laboratory Procedure, Alere) in a 40 μL blood sample, taken from a fingerstick. A second desktop point-of-care analyzer was used for measuring glycated hemoglobin A1c (HbA1c) through boronate affinity method, in a blood sample of 4 μL, drawn from the same fingerstick (Quo-Lab™ A1c, EKF Diagnostics).

### CVD and cardiovascular risk factors

Established cardiovascular disease (history of stroke or coronary artery disease) and cardiovascular (CV) risk factors (DM, hypercholesterolemia and AH) were diagnosed on the basis of patient reporting or/and referred medical history or/and relative medication regimen. Unknown diabetes (screen-detected) was diagnosed according to WHO criteria, based on either a fasting serum glucose value equal or higher to 7.0 mmol/l (126 mg/dl) or an HbA_1c_ value equal or higher to 6.5% [19, 20]. The cut-off values for diagnosing pre-diabetes were based on WHO criteria as well, i.e., fasting serum glucose between 5.6 and 6.9 mmol/l (100–125 mg/dl) or/and HbA_1c_ between 5.7 and 6.4%. Unknown AH (screen-detected) was diagnosed when at least two measurements of systolic or/and diastolic arterial pressure were equal or higher to 140 mmHg and/or 90 mmHg respectively. Additionally, unknown hypercholesterolemia was considered when serum values of total cholesterol were equal or higher to 5.2 mmol/l (200 mg/dl). Finally, metabolic syndrome was diagnosed based on the National Cholesterol Education Program Adult Treatment Panel III (2001) definition [21].

### Statistical analysis

We used PASW Statistics 19.0 (SPSS, Inc., Chicago, Illinois) for all statistical analyses. Continuous variables are expressed as mean ± standard deviation or median [interquartile range] and categorical variables are expressed as counts and percentages. In order to investigate for associations between adherence to the Mediterranean diet and other variables, linear regression analysis (both univariable and multivariable) was used, with the MedDietScore as the dependent variable. The variables included in the multivariable analysis were those found to be significant in the univariable model, as well as variables with known/putative association with dietary habits based on the literature. The level of statistical significance for inclusion was set at a *p* value of 0.05 or less. A stepwise backward elimination procedure was used in order to include the significant variables in the multivariable model. In order to test for differences in the frequency of food item consumption between groups, Pearson’s chi-square test was used. All significance tests were 2-tailed, and *p* <0.05 was considered to be statistically significant.

## Results

### Demographic and anthropometric characteristics

The initial study population included 612 individuals (76.5% of the island’s target population), who agreed to participate and signed an informed consent. Out of the initial population, 596 individuals (97.4%) provided fully completed questionnaires and underwent full clinical and laboratory assessment and, hence, are reported in the present analysis. The basic demographic and anthropometric characteristics are shown in Table 1. There were 287 males (48.2%) and the mean age of the population was 49.5 ± 19.6 years, with 164 individuals (27.5%) being older than 65 years old. The body mass index of the study participants, stratified by age and sex is shown in Table 2. Eighty five out of 287 men (29.6%) and 117/309 women (37.8%) were obese (BMI ≥ 30Kg/m^2^), while 26.8% of men and 29.7% of women had a BMI within the normal range (18.5–25.0 Kg/m^2^).

**Table 1:** Demographic and anthropometric characteristics of the study population, stratified by sex

Characteristic	Total	Mean ± SD or Count (%)		*P*
		Men	Women	
n	596	287 (48.2)	309 (51.8)	
Age (year)	49.4 ± 19.7	48.7 ± 19.9	50.0 ± 19.4	0.425
Height (cm)	162.9 ± 10.1	169.2 ± 8.2	157.1 ± 8.0	<0.001
Weight (kg)	74.9 ± 17.6	79.7 ± 18.0	70.4 ± 16.1	<0.001
Body Mass Index (kg/m^2^)	28.2 ± 6.2	27.8 ± 5.6	28.6 ± 6.6	0.107
Waist circumference (cm)	95.4 ± 16.2	97.9 ± 16.5	93.0 ± 15.6	<0.001
Hip circumference (cm)	104.6 ± 12.0	102.6 ± 10.5	106.6 ± 13.0	<0.001
Waist to hip ratio	0.91 ± 0.10	0.95 ± 0.10	0.87 ± 0.09	<0.001
Neck circumference (cm)	37.6 ± 4.5	40.2 ± 3.9	35.2 ± 3.6	<0.001
Total body fat (%)	30.2 ± 10.4	24.3 ± 8.5	35.7 ± 9.0	<0.001
Lean body mass (kg)	51.4 ± 10.7	59.3 ± 9.3	44.0 ± 5.4	<0.001
Education				
Illiterate/Few grades of elementary school	81 (13.6)	36 (12.5)	45 (14.6)	0.543
Elementary school graduate	198 (33.2)	92 (32.0)	106 (34.3)	
Three grade high school graduate	88 (14.7)	48 (16.7)	40 (12.9)	
Six grade high school graduate	153 (25.7)	78 (27.2)	75 (24.3)	
College/University graduate	76 (12.8)	33 (11.5)	43 (13.9)

**Table 2:** Counts and percentages of body mass index groups stratified by age and sex^a^

Age category (years)	Underweight	Normal weight	Overweight	Obese	Morbidly obese	Total
Men	8 (2.8)	77 (26.8)	117 (40.8)	76 (26.5)	9 (3.1)	287
< 35	7 (9.0)	42 (53.8)	19 (16.5)	8 (10.5)	2 (2.6)	78
35–65	0 (0)	23 (16.8)	61 (44.5)	46 (33.6)	7 (5.1)	137
> 65	1 (1.4)	12 (17.4)	35 (50.0)	22 (31.4)	0 (0)	70
Women	8 (2.6)	92 (30.0)	90 (29.5)	99 (32.2)	18 (5.9)	307
< 35	8 (10.1)	44 (55.7)	18 (22.8)	6 (7.6)	3 (3.8)	79
35–65	0 (0)	42 (28.6)	49 (33.3)	43 (29.3)	13 (8.8)	147
> 65	0 (0)	6 (7.4)	23 (28.4)	50 (61.7)	2 (2.5)	81

^a^Percentages are calculated for each row separately

BMI categories: Underweight: <18.5 kg/m^2^

Normal weight: 18.5–24.99 kg/m^2^

Overweight: 25–29.99 kg/m^2^

Obese: 30–39.99 kg/m^2^

Morbidly obese: ≥40 kg/m^2^

### History of CVD and CVD risk factors

A history of coronary artery disease (CAD) or stroke was reported by 6.4 and 2.0% of the participants, respectively. The prevalence of CVD risk factors (AH, DM and hypercholesterolemia), both known and screen detected, is depicted in Table 3.

**Table 3:** Prevalence of reported and screen detected classic cardiovascular risk factors stratified by sex^a^

Disease	Known	Medication-treated	Screen detected	Total
Diabetes mellitus	46 (7.7)	37 (6.2)	24 (4.0)	70 (11.7)
Men	20 (7.0)	16 (5.6)	15 (5.2)	35 (12.2)
Women	26 (8.4)	21 (6.8)	9 (2.9)	35 (11.3)
Arterial hypertension	184 (30.9)	171 (28.7)	77 (12.9)	261 (43.8)
Men	85 (29.6)	80 (27.9)	48 (16.7)	133 (46.3)
Women	99 (32.0)	91 (29.6)	29 (9.4)	128 (41.4)
Hypercholesterolemia	184 (30.9)	143 (24.0)	139 (23.3)	323 (54.2)
Men	72 (25.1)	59 (20.6)	67 (23.3)	139 (48.4)
Women	112 (36.2)	84 (27.4)	72 (23.3	184 (59.5)

^a^Numbers represent counts (percentages)

One hundred and eighty four individuals (30.9%) reported a history of AH, among whom one hundred and seventy one (93%) received anti-hypertensive medications. The most common regimen was the combination of angiotensin II receptor blocker (ARB) and a diuretic (21.4%), followed by an ARB (9.8%), a b-blocker (8.1%) and an angiotensin converting enzyme inhibitor monotherapy (8.2%). Fifty three (30.6%) patients received a calcium channel blocker, but this was rarely used as monotherapy (1.7%). Among those who did not report a history of hypertension, 77 participants (12.9% of the whole population) had screen-detected hypertension, raising the total prevalence to 43.8%.

Forty six individuals (7.7%) reported a history of DM, among whom 37 (80.4%) received a glucose-lowering medication. The most frequently administered drug regimens were metformin alone (43.2%) or in combination with a dipeptidyl peptidase 4 (DPP4) inhibitor (24.3%), while 10.8% received insulin therapy. An additional 24 individuals (4.0% of the total population) were diagnosed with diabetes during the study screening, raising the total prevalence to 11.7%. Pre-diabetes was detected in 139 participants (23.3%), among whom 110 were diagnosed according to fasting plasma glucose and 16 according to HbA1c levels and 13 by both methods.

One hundred and eighty four participants (30.9%) reported a history of hypercholesterolemia with 143 receiving lipid lowering therapy; statin alone (77.6%) or combined with ezetimibe (16.1%) were the most commonly used regimens. An additional 139 individuals had abnormally high levels of total cholesterol, based on the measurements during the study screening, raising the total prevalence of hypercholesterolemia to 54.2%. The rates of reported and screen-detected CVD risk factors stratified by sex and BMI are presented in Table 4.

**Table 4:** Prevalence of reported and screen detected classic cardiovascular risk factors stratified by body mass index and sex^a^

Disease	Known	Screen detected
Diabetes mellitus		
Men		
Underweight	0 (0)	0 (0)
Normal	2 (2.6)	3 (3.9)
Overweight	6 (5.1)	7 (6.0)
Obese	11 (14.5)	14 (18.5)
Morbidly obese	1 (11.1)	2 (22.2)
Women		
Underweight	0 (0)	0 (0)
Normal	2 (2.2)	3 (3.3)
Overweight	4 (4.4)	6 (6.6)
Obese	18 (18.2)	9 (9.1)
Morbidly obese	1 (5.6)	1 (5.6)
Arterial hypertension		
Men		
Underweight	1 (12.5)	1 (12.5)
Normal	8 (10.4)	16 (20.8)
Overweight	39 (33.3)	40 (34.2)^+^
Obese	35 (46.1)	30 (39.5)
Morbidly obese	2 (22.2)	3 (33.3)
Women		
Underweight	0 (0)	0 (0)
Normal	6 (6.5)	13 (16.3)
Overweight	32 (35.2)	17 (19.1)^+^
Obese	51 (51.5)	45 (45.9)
Morbidly obese	9 (50.0)	8 (50)
Hypercholesterolemia		
Men		
Underweight	1 (12.5)	0 (0)
Normal	6 (7.8)	23 (29.9)^+^
Overweight	41 (35.0)	47 (40.4)
Obese	23 (30.3)^+^	37 (48.7)
Morbidly obese	1 (11.1)	7 (77.8)
Women		
Underweight	0 (0)	0 (0)
Normal	11 (12.0)	44 (47.8)^+^
Overweight	42 (46.2)	34 (37.8)
Obese	53 (53.5)^+^	34 (34.3)
Morbidly obese	5 (27.8)	11 (64.7)

^a^Numbers represent counts (percentages)

BMI categories: Underweight: <18.5 kg/m^2^

Normal weight: 18.5–24.99 kg/m^2^

Overweight: 25–29.99 kg/m^2^

Obese: 30–39.99 kg/m^2^

Morbidly obese: ≥40 kg/m^2^

^+^ *P* < 0.05

Regarding metabolic syndrome, its diagnosis was set in 208 (34.9%) individuals; 102 (35.5%) men and 106 (34.3%) women. The prevalence of metabolic syndrome was 121/444 (27.3%) in individuals ≤ 65 years old and 87/152 (57.2%) in individuals >65 years old.

### Lifestyle and dietary habits

Two hundred and eighteen individuals (36.6%) were active smokers with a mean smoking burden of a median 18 [27.5] pack-years, whereas 90 (15.1%) were former smokers with a median time since cessation of 15 [20] years. The mean duration of sleep during the night was 7.5 ± 1.9 h, while the proportion of individuals who slept systematically during mid-day was 57.2%. With regards to physical activity, 72.5% of the individuals reported some kind of regular physical activity during the week, while 27.5% reported that they are physically inactive. Walking was the most commonly reported type of activity (73.0% of cases).

**Fig. 2:**
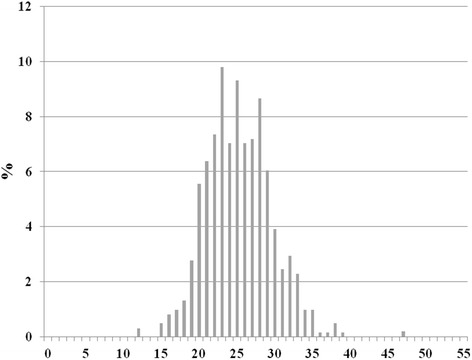
Distribution of MedDietScore values among the study participants

The frequency at which the various items of the dietary questionnaire were consumed by the study participants are depicted in Table 2 of the Additional file 1. Olive oil was consumed on a daily basis by virtually all individuals (96.2%). Fruits and vegetables were consumed on a daily basis by 34.7% and 35.9% respectively, while fish was consumed at least three times per week by 46.3%. Two hundred and seventy participants (45.3%) consumed alcohol on a regular basis, with a median of 2 [3.5] glasses per day, respectively. The mean and the median value of the MedDietScore were 25.3 ± 4.4 and 25 [6] respectively. The distribution of MedDietScore values in the study sample is shown in Fig. 2.

In a further analysis of between sex differences in dietary habits with the use of Chi-square test, men demonstrated less frequent (*P* = 0.038) consumption of vegetables and full-fat dairy products (*P* = 0.012), but more frequent consumption of red meat (*P* = 0.034), fish (*P* = 0.001) and alcohol (*P* < 0.001).

Results of the univariable and multivariable analysis examining factors associated with higher adherence to the Mediterranean diet are shown in Table 5. The variables that were shown to be significant in the univariate analysis and were included in the backward step multivariate linear regression test were age, sex, mid-day sleep, presence of physical activity, history of hypertension, hypercholesterolemia and coronary artery disease, waist circumference, arterial blood pressure and heart rate. Furthermore, history of diabetes mellitus and level of education, which are factors known to affect quality of diet were also included in the multivariate analysis. Hence, at the end, age (β:0.067; 95% confidence interval [CI]: 0.044-0.091, *P* < 0.001), male sex (β:2.199; 95% CI: 1.488-2.909, *P* < 0.001), history of hypercholesterolemia (β: 0.906; 95% CI: 0.026-1.787, *P* = 0.044), history of diabetes (β: −1.431; 95% CI: −2.772-[−0.091], *P* = 0.036), physical activity (any vs. no activity) (β: 1.307; 95% CI: 0.517-2.097, *P* = 0.001) and level of education (β: 0.407; 95% CI: 0.099-0.715, *P* = 0.010) were the factors independently associated with a higher dietary score and consequently a better adherence to the Mediterranean diet patterns.

**Table 5:** Univariate and multivariate linear regression for participants’ baseline characteristics and study measurements as determinants of dietary score

Characteristic	Univariate Analysis		Multivariate Analysis	
	β (95% CI)	*P* Value	β (95% CI)	*P* Value
Age, y	0.05 (0.032-0.068)	<0.001	0.067 (0.044-0.091)	<0.001
Male sex, %	2.068 (1.362-2.773)	<0.001	2.199 (1.488-2.909)	<0.001
Sleep duration, h	−0.023 (−0.217-0.170)	0.815		
Mid-day sleep, %	0.765 (0.034-1.496)	0.040		
Snoring, %	0.401 (−0.332-1.135)	0.283		
Level of education, %	−0.028 (−0.316-0.260)	0.847	0.407 (0.099-0.715)	0.010
Active smoking, %	0.203 (−0.550-0.955)	0.597		
Physical activity, %	1.075 (0.263-1.888)	0.010	1.307 (0.517-2.097)	0.001
Family history of CVD, %	0.083 (−0.174-0.339)	0.526		
History of hypertension, %	1.124 (0.346-1.903)	0.005		
History of hypercholesterolemia, %	1.250 (0.468-2.032)	0.002	0.906 (0.026-1.787)	0.044
History of diabetes, %	−0.503 (−1.876-0.871)	0.472	−1.431 (−2.772-[−0.091])	0.036
History of coronary artery disease, %	2.064 (0.562-3.566)	0.007		
Waist circumference, cm	0.038 (0.015-0.060)	0.001		
Body mass index, kg/m^2^	0.048 (−0.011- 0.106	0.109		
Systolic arterial pressure, mmHg	0.025 (0.009-0.041)	0.003		
Heart rate, bpm	−0.033 (−0.061- -0.005)	0.022		
Serum creatinine, mg/dl	0.253 (−0.615-1.122)	0.565		

## Discussion

The current study demonstrates that obesity, diabetes, hypertension and hypercholesterolemia are highly prevalent among the inhabitants of the small, relatively isolated Greek island of the Aegean Sea, Elafonisos. Moreover, a large proportion of the participants were unaware of their condition.

The findings of our study are in line with epidemiologic studies repeatedly showing a steep increase of obesity and CVD risk factors worldwide during the last 40 years [22, 23]. Moreover, the prevalence of overweight and obesity in our study approximates the figures reported by a large cross-sectional nationwide survey almost a decade ago [24]. As expected the prevalence of overweight and obesity increases significantly after the age of 45 years in both sex groups. However, in contrast to the previously reported Greek data, obesity seems to be more common among women inhabitants of Elafonisos. This distinct finding could be attributed to the special occupational characteristics of this population, as many of the male inhabitants of the island are engaged in agricultural, farming and piscatorial activities, whereas many of the female inhabitants are mainly occupied in housework activities.

The prevalence of reported DM in our study (7.7%) was comparable to the one of drug-prescribed DM among Greek people > 15 years of age (8.2%), found in a recent, nationwide prescription database study [25]. However, it was significantly lower than the one reported (12.0%) in a recent cross-sectional survey of a randomly selected population of the municipality of Saronikos [26], which is part of the greater Athens area, These differences among individual populations may reflect the effect of genetic, environmental or health policy factors. Finally, with regards to arterial hypertension, our results are in line with those reported by the ATTICA study in 2001 [27]. The prevalence of AH seems to be rapidly increasing among the Greek population and its prevalence is consistently higher in men than women [27].

Observational studies from as early as the 1990’s have outlined a subtle, though apparent overtime trend towards an unhealthier lifestyle adaptation among the Greek population. In a study performed in 1995, Voukiklaris et al. reported increased prevalence of CVD risk factors, including hypertension, dyslipidemia and smoking, accompanied by a respective decrease in heavy physical activity and an impressively higher incidence of CVD in middle-aged men from rural areas of the island of Crete [28]. Later studies from the same geographical area reported increased prevalence of overweight and obesity among farmers; mean BMI was an impressive 7 kg/m^2^ higher when compared to the cohort of the SCS from 40 years earlier [29]. This change was discussed by the authors as being attributable to the gradual establishment of a sedentary life-style, away from high-intensity physical activity and the adoption of unfavorable dietary habits (increased intake of meat and saturated fat, and decrease in fruit consumption) [30, 31], despite the fact that the caloric intake surprisingly decreased overtime [32, 33]. The island of Crete has been in the spotlight of several epidemiologic studies due to the fact that it provided the initial cohort of the SCS, based on which the concept of the “Mediterranean diet” was perceived and established. On the other hand, Crete (the 5^th^ largest island of the Mediterranean Sea and the largest island in Greece, having approximately 620,000 permanent inhabitants in 2011), includes large urban centers with several daily connections by airplane and ship to the mainland. Consequently, its epidemiological and geographical characteristics are in no case representative of the population of the Greek islands in general.

In the present study population, although the participants reported some dietary habits representative of the traditional Mediterranean diet, such as the everyday consumption of olive oil or the frequent consumption of fish, they also seem to have adopted several aspects of “Westernized” diet, such as the limited consumption of fruits and vegetables and the frequent consumption of meat and sweets. Overall and according to their median MedDietscore (=25), their habits may be characterized as moderately adherent to the Mediterranean diet. Importantly, a strong, independent association was designated between age and adherence to Mediterranean diet in our population. This finding can be potentially attributed to the changes in lifestyle and occupation which have been observed during the past two decades due to the touristic development of the island. In addition, the island population demonstrated a rather sedentary life-style, with complete lack of physical activity in approximately one fourth of the participants, while only 49% reported some form of physical activity on a daily basis. Interestingly, the participants who exercised reported a more healthy dietary behavior, as physical activity along with age, male sex, level of education, history of hypercholesterolemia and lack of history of diabetes were independently associated with higher adherence to the Mediterranean diet. The association between age and healthier dietary habits has been further corroborated by the findings of another study that highlights age as an independent predictor of adherence to Mediterranean diet in a large cohort of individuals from four randomly selected Greek islands [34]. This could be explained not only by the lower caloric requirements of the elderly population, but, additionally, by their increased resistance in adopting newly introduced “Westernized” dietary and life-style trends. Moreover, one must bear in mind that the greater adherence of the elderly to Christian Orthodox rituals, among which is frequent fasting, may in part explain their healthier eating habits, as previously discussed [35–37].

The association between history of hypercholesterolemia and higher adherence to the Mediterranean diet comes as no surprise, since dietary recommendations against this condition are compatible to the Mediterranean diet patterns, namely increasing intake of vegetables, fruits, and whole grains, as well as reduced intake of sweets, sugar-sweetened beverages and red meat. On the other hand, since the same dietary recommendations also apply for patients with diabetes, the inverse relationship depicted between history of diabetes and adherence to the Mediterranean diet in our population could possibly indicate either the limited access of the Elafonisos inhabitants to specialized, in the field, physicians or lack of adequate compliance on their behalf. The association between male sex and better adherence to the Mediterranean diet is contradictory to previous results [16, 36]. However, in the present study, this sex difference was driven by an increased intake of alcohol and fish among men, possibly indicating the special social and occupational characteristics of the study’s population. Furthermore, the positive relationship between level of education and adherence to Mediterranean diet comes as no surprise. Education, as a component of higher socioeconomic status, has been shown to correlate inversely with the incidence of CVD. The same findings were confirmed in a recent sub-analysis of the ATTICA population, which showed a significantly higher 10-year incidence of CVD among individuals with lower (≤9 years) vs higher education (≥15 years) [38]. This positive effect can in part be attributed to the greater ease of access to edifying and behavior-modifying information and programs that education ensures. Additionally, higher education is in general paralleled by higher income; this enables better access to higher quality health facilities.

The notably high prevalence of obesity and both known and screen-detected CVD risk factors places the study population at high risk for incident CVD. The relatively low prevalence of reported CVD (8.4%) among this population merits further investigation, as it could reflect either genetic factors or possible beneficial effects of certain aspects of the Mediterranean diet, such as olive oil and fish consumption or a misinterpretation due to the latent period of CVD. Increased inbreeding, which is frequent among isolated populations, reinforces the possibility that genetic factors may play a pivotal role in the disequilibrium between prevalence of CVD and risk factors in our study population. However, similar trends have been reported in the past among the Greek population. In the ATTICA study, the baseline prevalence of known CVD was also low compared to the widely prevalent CVD risk factors [27]. However, CVD incidence during the 5- and 10-year follow-up was as high as 8.5 and 15.7%, respectively [39], raising the total burden of the disease very fast. Consequently, only prospective evaluation during long-term follow-up will help to elucidate the association between risk factors and CVD in this particular population.

### Study limitations

Due to the cross-sectional nature of this analysis and the single island population included, generalization of the results should be applied with caution before confirmation is available from larger population analyses. However, this does not diminish the significance of our findings, since they clearly show that the inhabitants of a small island of the Mediterranean basin, have adopted “Westernized” lifestyle habits. Although, the cross-sectional analysis does not allow deduction of causal relationships between the study variables, the fact that overweight, obesity and CVD risk factors are highly prevalent among this population cannot be considered a coincidence.

Although other measures of body composition assessment, such as CT scan and MRI are more accurate than bio-impendance, the latter, was used in due to its simplicity, quickness, low cost and non-invasive nature. Bio-impedance can accurately measure body fat, as far as it is used for specific ethnic groups, populations and conditions, which was actually the case in the present study.

Finally, although a single measurement of fasting serum glucose for the diagnosis of diabetes is subject to error, in epidemiological studies, one measurement is most often used, due to infeasibility of double measurements. In the present study, individuals with high blood glucose results (in the range of diabetes) were advised to show them to their family physicians in order to receive further instructions.

## Conclusions

In summary, the prevalence of obesity and classical CVD risk factors is high among the inhabitants of a small Eastern Mediterranean island, showing that even isolated rural populations have adapted many aspect of the “Western” lifestyle. This increase, combined with distancing of the population from traditional dietary and life-style habits should ring a bell for all health policy makers; organized, multi-faceted efforts should be made to promote re-adoption of the Mediterranean diet in an attempt to reduce the burden of CVD and improve population’s health.

## Additional file

Additional file 1:
**Table 1**. MedDietScore points according to frequency of consumption of different food item categories. **Table 2**. Frequency of consumption of different food item categories (DOCX 17 kb)
Additional file 2: PERSEAS population raw data. (XLSX 194 kb)

## Abbreviations

AH: Arterial hypertension
ARB: Angiotensin II receptor blocker
BMI: Body mass index
CAD: Coronary artery disease
CI: Confidence interval
CVD: Cardiovascular disease
DM: Diabetes mellitus
DPP4: Dipeptidyl peptidase-4
HbA1c: Glycated hemoglobin A1c
PERSEAS: Prospective Evaluation of cardiovascular Risk Surrogates in Elafonisos Area Study
SCS: Seven Country Study
