# Supplementary material for: Diet, life-style and cardiovascular morbidity in the rural, free living population of Elafonisos island
Source: BMC Public Health. 2017 Feb 1;17:147. doi: 10.1186/s12889-017-4053-x (PMC5286858; doi:10.1186/s12889-017-4053-x)
Supplement: Additional file 1: — Table 1. MedDietScore points according to frequency of consumption of different food item categories. Table 2. Frequency of consumption of different food item categories (DOCX 17 kb) [file 12889_2017_4053_MOESM1_ESM.docx]

**APPENDIX**

**Table 1. MedDietScore points according to frequency of consumption of different food item categories**

| **Dietary item** | **Frequency** | | | | | |
| --- | --- | --- | --- | --- | --- | --- |
|  | **<1 time per week** | **1-3 times per week** | **4-6 times per week** | **Once daily** | **2-3 times per day** | **>4 times per day** |
| Whole grain cereal | 0 | 1 | 2 | 3 | 4 | 5 |
| Potatoes | 0 | 1 | 2 | 3 | 4 | 5 |
| Fruits | 0 | 1 | 2 | 3 | 4 | 5 |
| Vegetables | 0 | 1 | 2 | 3 | 4 | 5 |
| Full fat dairy products | 5 | 4 | 3 | 2 | 1 | 0 |
|  | **Never** | **<1 time per week** | **1-2 times per week** | **3-4 times per week** | **5-6 times per week** | **>6 times per week** |
| Red meat and its products | 5 | 4 | 3 | 2 | 1 | 0 |
| Poultry | 5 | 4 | 3 | 2 | 1 | 0 |
| Legumes | 0 | 1 | 2 | 3 | 4 | 5 |
| Fish | 0 | 1 | 2 | 3 | 4 | 5 |
| Olive oil | 0 | 1 | 2 | 3 | 4 | 5 |
|  | **None or > 700 ml per day** | **<700 ml per day** | **<600 ml per day** | **400-500 ml per day** | **300 ml per day** | **<300 ml per day** |
| Alcohol | 0 | 1 | 2 | 3 | 4 | 5 |

**Table 2. Frequency of consumption of different food item categories***

| **Dietary item** | **Frequency** | | | | | |
| --- | --- | --- | --- | --- | --- | --- |
|  | **<1 time per week** | **1-3 times per week** | **4-6 times per week** | **Once daily** | **2-3 times per day** | **>4 times per day** |
| Whole grain cereal | 302 (50.7) | 160 (26.8) | 60 (10.1) | 58 (9.7) | 13 (2.2) | 3 (0.5) |
| Potatoes | 37 (6.2) | 288 (48.3) | 238 (39.9) | 26 (4.4) | 5 (0.9) | 2 (0.3) |
| Fruits | 89 (14.9) | 163 (27.3) | 137 (23.0) | 151 (25.3) | 52 (8.7) | 4 (0.7) |
| Vegetables | 36 (6.0) | 159 (26.7) | 187 (31.4) | 198 (33.2) | 15 (2.5) | 1 (0.2) |
| Full fat dairy products | 28 (4.7) | 89 (14.9) | 179 (30.0) | 158 (26.5) | 126 (21.3) | 16 (2.7) |
|  | **Never** | **<1 time per week** | **1-2 times per week** | **3-4 times per week** | **5-6 times per week** | **>6 times per week** |
| Red meat and its products | 31 (5.2) | 139 (23.3) | 275 (46.1) | 122 (20.5) | 25 (4.2) | 4 (0.7) |
| Poultry | 22 (3.7) | 68 (11.4) | 323 (54.2) | 152 (25.5) | 28 (4.7) | 3 (0.5) |
| Legumes | 28 (4.7) | 112 (18.8) | 416 (69.8) | 39 (6.5) | 1 (0.2) | 0 (0) |
| Fish | 23 (3.9) | 63 (10.6) | 233 (39.1) | 151 (25.3) | 105 (17.6) | 20 (3.4) |
| Olive oil | 0 (0) | 0 (0) | 1 (0.2) | 12 (2.0) | 9 (1.5) | 564 (96.2) |
|  | **None or > 700 ml per day** | **<700 ml per day** | **<600 ml per day** | **400-500 ml per day** | **300 ml per day** | **<300 ml per day** |
| Alcohol | 343 (57.6) | 1 (0.2) | 4 (0.7) | 25 (4.2) | 10 (1.7) | 213 (35.7) |
